# Transient interactions between the fuzzy coat and the cross-β core of brain-derived Aβ42 filaments

**DOI:** 10.1126/sciadv.adr7008

**Published:** 2025-01-15

**Authors:** Maria Milanesi, Z. Faidon Brotzakis, Michele Vendruscolo

**Affiliations:** ^1^ Centre for Misfolding Diseases, Department of Chemistry, University of Cambridge, Cambridge CB2 1EW, UK.; ^2^ Unit of Macromolecular Interaction Analysis, Department of Molecular and Translational Medicine, University of Brescia, 25123 Brescia, Italy.; ^3^ Institute for Biomedical Technologies, National Research Council (ITB-CNR), 20054 Segrate (MI), Italy.; ^4^ Institute for Bioinnovation, Biomedical Sciences Research Center “Alexander Fleming”, 16672 Vari, Greece.

## Abstract

Several human disorders, including Alzheimer’s disease (AD), are characterized by the aberrant formation of amyloid fibrils. In many cases, the amyloid core is flanked by disordered regions, known as fuzzy coat. The structural properties of fuzzy coats, and their interactions with their environments, however, have not been fully described to date. Here, we generate conformational ensembles of two brain-derived amyloid filaments of Aβ42, corresponding respectively to the familial and sporadic forms of AD. Our approach, called metadynamic electron microscopy metainference (MEMMI), provides a characterization of the transient interactions between the fuzzy coat and the cross-β core of the filaments. These calculations indicate that the familial AD filaments are less soluble than the sporadic AD filaments, and that the fuzzy coat contributes to solubilizing both types of filament. These results illustrate how the metainference approach can help analyze cryo-EM maps for the characterization of the properties of amyloid fibrils.

## Teaser

The combination of molecular simulations and cryo-EM density maps reveal the dynamics of brain-derived Abeta42 filaments.

## INTRODUCTION

Alzheimer’s disease (AD) causes cognitive impairment through neuronal loss and accounts for 60 to 80% of the cases of dementia worldwide (*1*, *2*). The accumulation of intracellular neurofibrillary tangles, formed by the tau protein, and of extracellular senile plaques, formed by the amyloid-β (Aβ) peptide, represents the main hallmark of the disease at the molecular level (*3*–*5*). In the amyloidogenic pathway, Aβ is generated through successive cleavages of the amyloid precursor protein (APP) by β-secretase and ɣ-secretase (*5*). Aβ is poorly soluble and readily aggregates into amyloid fibrils (*6*). The surface of the amyloid fibrils acts as a catalyst for secondary nucleation, with a feedback mechanism that facilitates the proliferation of the amyloid fibrils themselves (*6*, *7*).

The structures of amyloid fibrils are characterized by a distinctive molecular architecture known as cross-β (*6*, *8*). This architecture consists of β strands that stack perpendicularly to the long axis of the fibril, forming structures called protofilaments. Multiple protofilaments can then associate laterally, giving rise to twisted fibrils (*6*, *8*, *9*). Investigations of synthetic Aβ peptides in in vitro preparations revealed a spectrum of different polymorphic structures of Aβ fibrils (*10*–*14*). Furthermore, recent reports indicated that these fibrils are structurally different from amyloid fibrils derived from brain tissue (*15*–*17*). The cryo–electron microscopy (cryo-EM) structures of Aβ42 fibrils extracted from the parenchyma brain tissue of 10 patients with AD were recently reported (*17*). Two distinct polymorphs were distinguished, referred to as, respectively, type I filaments, principally found in sporadic AD (sAD), and type II filaments, present in familial AD (fAD) and other conditions. The atomic model of the type I filament spans from residues G9 to A42, forming the organized structural core of the protofilament structure, which exhibits the characteristic cross-β architecture of amyloid fibrils. This type I filament consists of two S-shaped protofilaments closely packed together through hydrophobic interactions, exhibiting pseudo-2_1_ symmetry. In contrast, the cross-β core of the type II filament spans from residues V12 to A42, and it is made up of two protofilaments forming the fibril with a C2 symmetry. The structural models obtained from both filaments are missing about 20% of the Aβ42 sequence, represented by the N-terminal residues. This N-terminal regions is expected to remain disordered, displaying a high conformational heterogeneity, collectively forming a fuzzy coat around the cross-β core of the filaments (*18*–*21*). Further analysis of the structural properties of the disordered regions in the amyloid fibrils requires methods capable of characterizing conformational heterogeneity. The use of cryo-EM for this purpose remains quite challenging (*22*–*31*). Consequently, most structural studies on amyloid fibrils primarily concentrate on characterizing conformations and interactions within the cross-β cores, while the more dynamic flanking regions remain less explored.

The prominent role of the N-terminal region of Aβ42 fibrils in the aggregation process and the stability of the amyloid fibrils has been documented in several studies. This is substantiated by the observed influence of various N-terminal mutants on the aggregation process and the subsequent neurotoxic effects of the peptide (*32*–*36*). Nevertheless, our understanding of the impact of the fuzzy coat region on various functionalities and characteristics of the amyloid filaments—such as its interactions with the cross-β core, its contribution to the binding between the amyloid filaments and other biologically relevant components, and the solubility of the amyloid filaments—remains elusive. These insights would be of great importance for pharmacological research, as they could guide the development of new drugs and deepen our understanding of the molecular mechanisms of existing drugs targeting the N-terminal region of the filaments.

In this study, we report the structural ensembles of Aβ42 in the type I and type II filaments, obtained using the metadynamic electron microscopy metainference (MEMMI) method (*37*). The analysis of these structural ensembles provides insights into the role of the fuzzy coat in modulating the properties of amyloid fibrils.

## RESULTS

### Structure and dynamics of type I and type II filaments

**Fig. 1.**
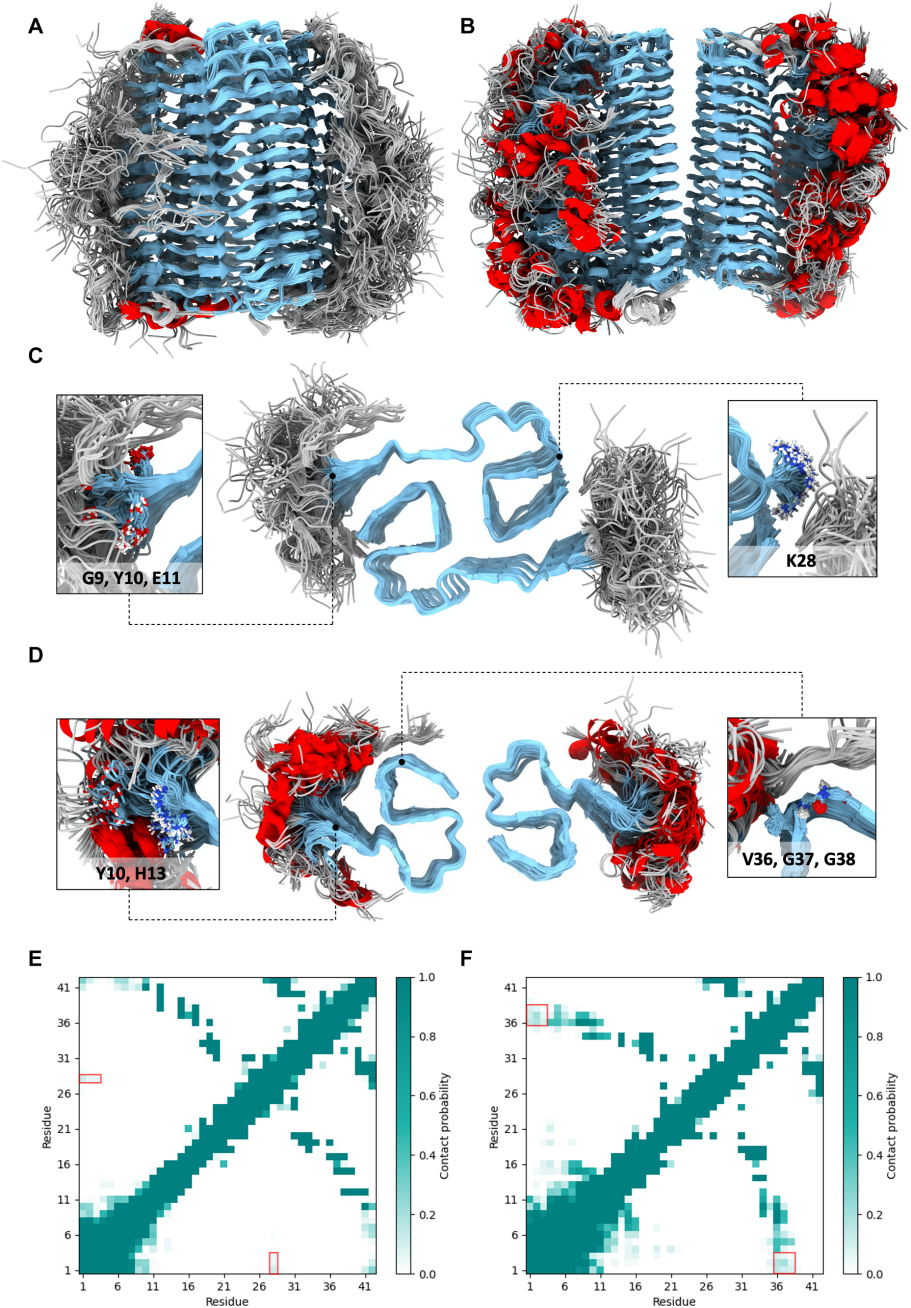
Structural ensembles of type I and type II filaments formed by Aβ42. (**A** and **B**) Structural ensembles of type I (A) and type II (B) filaments, obtained by extracting 100 conformations from the structural ensembles generated in this work. (**C** and **D**) Lateral view of the structural ensembles and close-up of the residues shielded by the fuzzy coat of type I (C) and type II (D) filaments. The colors denote secondary structure elements: β sheet (cyan), coil (gray), and α helix (red). The insets show residues G9, Y10, E11, and K28 in type I filaments and residues Y10, H13, V36, G37, and G38 in type II filaments. These residues are on the surface of the cross-β core but are partially shielded from the solvent by interactions with the fuzzy coat. (**E** and **F**) Contact maps corresponding to the structural ensembles of type I (E) and type II (F) filaments. The contact maps show the contact probability between residue pairs. Contacts with probability 1 (teal) are always formed, contacts with probability 0 (white) are never formed, and contacts with intermediate probabilities are transient. As examples the red boxes highlight the transient contacts between residues D1, A2, and E3 with residue K28 (E) and between residues D1, A2, and E3 with residues V36, G37, and G38 (F).

The structures of the cross-β cores of Aβ42 filaments derived from AD brains were recently reported by cryo-EM (*17*). These cross-β cores exhibit two types of structurally related protofilaments, resulting in the formation of two distinct filament types. Type I filaments were predominantly found in the brains of individuals with sAD [Protein Data Bank (PDB): 7q4b, EMD-13800], whereas type II filaments (PDB: 7q4m, EMD-13809) were primarily observed in the brains of individuals with fAD and other misfolding-related pathologies. In this study, we determine the structural ensemble of the whole Aβ42 filaments (residues 1 to 42) using the MEMMI method (*37*). Multiple collective variables (CVs) were biased using metadynamics (*38*, *39*) to accelerate the conformational sampling. Diffusion traces of the CVs and the free energy profiles as a function of simulation time and the block averaging analysis for each CV trace indicate that the simulations are converged (figs. S1 to S3). We modeled the system as a stack of 12 peptides on each side. Within the cross-β core, a parallel β sheet structure is consistently maintained. By contrast, we observed high conformational heterogeneity in the N-terminal for both type I (residues 1 to 9) (Fig. 1, A and C) and type II (residues 1 to 12) (Fig. 1, B and D) filaments. The resulting conformational ensembles can be described as a fuzzy coat that surrounds the cross-β core of the filaments.

### Secondary structure of the fuzzy coat and cross-β core

**Fig. 2.**
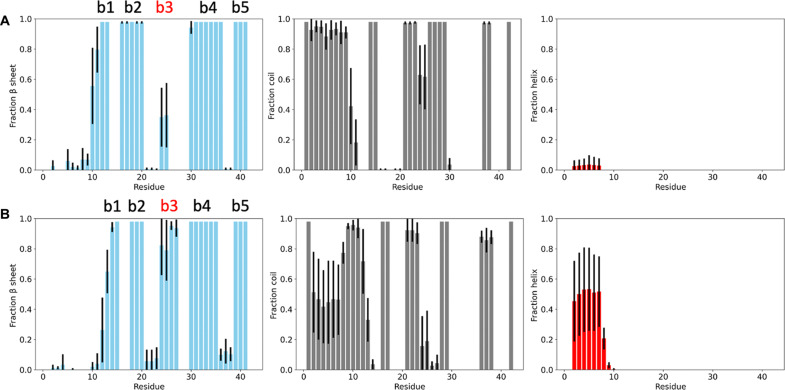
Secondary structure populations of the structural ensembles of type I and type II filaments. (**A** and **B**) Type I filaments (A) and type II filaments (B). Error bars are calculated as SDs between the first and second halves of the MEMMI simulations. The colors are based on the secondary structure elements: β sheet (cyan), coil (gray), and α helix (red).

Within the cross-β core, our findings support the β-sheet composition originally reported (*17*). Our calculations reveal an additional transient short β motif at residues 24 to 25 in the type I filament (Fig. 2A, left). Similar trends are also observed in the type II filament (Fig. 2B). Furthermore, our results indicate a higher α-helical content in the fuzzy coat of the type II filament (residues 2 to 8) compared to the type I filament, which exhibits a more coiled and disordered structure. These findings align with similar observations made in other amyloidogenic proteins, such as huntingtin, where the fuzzy coat adopts an α-helical structure, suggesting potential biologically relevant functions (*40*). We note that in type I filaments, the fuzzy coat appears more disordered, adopting a coiled structure, while type II filaments show a higher α-helical propensity in the fuzzy coat. This structural variation influences the interactions of the filaments with their environments. The ordered α-helical structure in type II fuzzy coats may provide increased stability and a scaffold for potential interactions with cellular membranes, metal ions, and other macromolecules. In contrast, the disordered fuzzy coat in type I filaments may contribute to higher solubility and different aggregation dynamics.

### Fuzzy coat interactions with the cross-β core

**Fig. 3.**
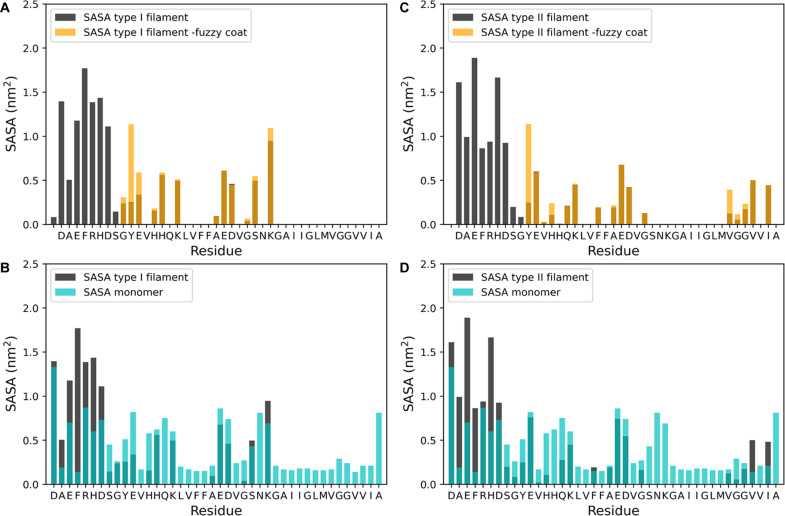
Effect of the fuzzy coat on the SASA for type I and type filaments. (**A** to **D**) Black bars represent the per residue SASA analysis of type I filament [(A) and (B)] and type II filament [(C) and (D)]. These SASA values were calculated on the basis of 100 conformations extracted from the MEMMI structural ensemble. Orange bars in (A) and (C) show the per residue SASA values of the 100 obtained structures after removing the N terminus. Light blue bars in (B) and (D) correspond to SASA values of the Aβ42 monomers in solution (*41*).

The fuzzy coat created by these N-terminal disordered regions extends to make contacts with the cross-β core. To investigate these contacts, we determined that the average distance between the fuzzy coat and the cross-β core, as quantified by the distribution of the CV ‘distance between the center of mass’ (DCM) (table S2), is approximately 3 nm for both filament types (fig. S4). Contact maps analysis reveals the transient nature of the interaction between the N-terminal and the cross-β core (Fig. 1, E and F), identifying specific residues that are transiently shielded by the dynamics of the fuzzy coat. Contact maps for each type of residue-residue interaction (hydrogen bonds, hydrophobic interaction, or salt bridge) were also calculated (fig. S5). To gain insights into how the fuzzy coat affects the structure and dynamics of the filaments, we performed a solvent-accessible surface area (SASA) analysis (Fig. 3). Comparing the SASA per residue of the structural ensemble of the complete filament structure with the SASA obtained when excluding the N terminus residues, we observed a SASA reduction for residues G9, Y10, E11, and K28 in type I filament and residues Y10, H13, V36, G37, and G38 for type II filament. This analysis, together with the previous described contact map results identified the specific residues that are shielded by the presence of the fuzzy coat during the filament dynamics (Figs. 1, C and D, and 3, C and D).

### Influence of the fuzzy coat on the solubility scores of type I and type II filaments

**Fig. 4.**
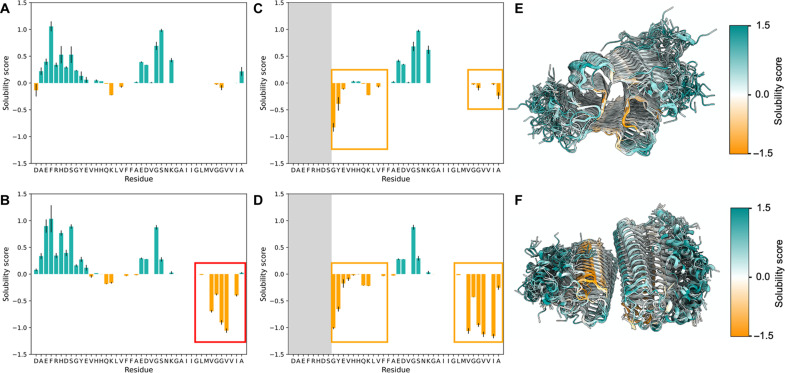
Influence of the fuzzy coat on the solubility scores of type I and type II filaments. (**A** and **B**) CamSol structurally corrected solubility score per residue for type I (A) and type II (B) filaments; mean and SDs are calculated from 20 randomly selected conformations from the MEMMI structural ensemble. Residues with solubility scores above 0 tend to be soluble, shown in light sea-green, while negative values indicate insoluble residues, shown in orange. The red square indicates the region of low solubility score in type II filaments. In type I filaments, this region is protected because it is buried in the core of the amyloid structure. (**C** and **D**) CamSol structurally corrected solubility scores per residue for type I (C) and type II (D) filaments; the same 20 conformations analyzed in (A) and (B) are shown, except that the fuzzy coat was removed. The orange squares indicate regions of low solubility score that are partially protected by the fuzzy coat. These conformations correspond to type I [(A) and (C)] and type II [(B) and (D)] filaments. (**E** and **F**) Graphical representation of the 20 conformations colored by the solubility score, with orange indicating less soluble regions and light sea-green indicating more soluble regions.

The comparison of the SASA of the whole structure of the two Aβ42 filaments types with respect to the SASA of the Aβ42 monomer in solution (*41*) indicates that the hydrophobic region in the C terminus region of the protein (*37*–*42*) is not exposed to the solvent in type I filaments (Fig. 3B), generating a more soluble filament (Fig. 4A), according to the structurally corrected CamSol solubility score (*42*, *43*). Instead in the type II filaments, it remains exposed to the solvent (Fig. 3D), generating a more insoluble filament (Fig. 4B, red box). This result is also confirmed by the electrostatic potential surface of the two filament types (fig. S6).

To explore the effects of the fuzzy coat on the solubility of the cross-β core, we calculated the structurally corrected CamSol solubility score obtained by excluding the fuzzy coat (Fig. 4, C to F). We calculated the average CamSol solubility score for the residues in the cross-β core for the type I and type II filaments. This calculation was performed for both the ensemble with the fuzzy coat present and the ensemble from which the fuzzy coat was removed (Table 1). The comparison of these solubility scores indicates that the fuzzy coat increases the solubility scores of the cross-β core of the filaments (Fig. 4, C and D, orange squares). This result is in accordance with the shielded residues observed in the contact map and SASA analysis reported above.

**Table 1.** Average solubility values with corresponding errors for the cross-β core of type I and type II Filaments. This table presents the averages of solubility values, along with their corresponding errors, for the cross-β core of type I and type II filaments. The values are derived from a 20 conformation ensembles, considering both ensembles with (+ fuzzy coat) and without (− fuzzy coat) the fuzzy coat.

	Type I filament	Type II filament
+ fuzzy coat	0.02 ± 0.01	−0.48 ± 0.07
− fuzzy coat	−0.05 ± 0.08	−0.71 ± 0.09

Our results indicate that type I and type II filaments exhibit distinct solvation properties, with type I filaments generally being more soluble than type II filaments. This increased solubility in type I filaments arises from the burial of the hydrophobic C-terminal region within the cross-β core, reducing its exposure to the solvent. In type II filaments, this region remains exposed, decreasing the solubility of the filament. These differences may have implications for the pathology of AD. More soluble filaments (type I) may spread differently in the brain and potentially interact less with surrounding cellular components compared to the more insoluble type II filaments, which are associated with fAD and may form more stable deposits in brain tissues.

### Correlation between experimental and calculated cryo-EM maps

**Fig. 5.**
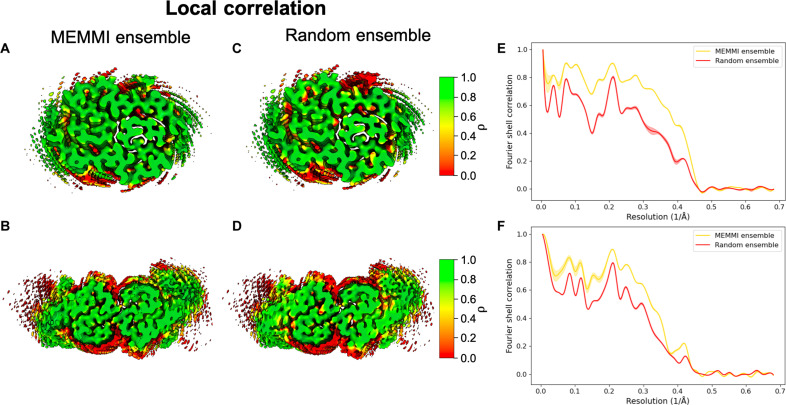
Local correlations and FSC curves of the MEMMI simulations. (**A** to **D**) Assessment of the local correlation between the cryo-EM map EMD-13800 [(A) and (C)] and EMD-13809 [(B) and (D)] with the cryo-EM maps back-calculated of the respective MEMMI structural ensemble [(A) and (B)] and for the random ensemble [(C) and (D)]. (**E** and **F**) FSC curves of type I (E) and type II (F) filaments for the density map generated by the random ensemble against the experimental cryo-EM map in red and for the map generated by the MEMMI ensemble against the experimental cryo-EM map in yellow. The lines represent the mean curves derived from the average of three ensembles for both MEMMI and RANDOM datasets, and the SD is represented as shaded areas.

To assess the fidelity of the MEMMI structural ensembles in capturing the molecular behavior and dynamics of type I and type II filaments, we examined their correlation with experimental cryo-EM maps (Fig. 5). The MEMMI ensembles for type I (Fig. 5A) and type II (Fig. 5B) filaments showed correlation coefficients with the experimental cryo-EM maps of 0.91 and 0.88, respectively. For comparison, we generated ensembles for both filament types with N-terminal structures in random conformations, resulting in correlation coefficients with the experimental cryo-EM maps of 0.79 and 0.77 (Fig. 5, C and D), respectively. In addition, we calculated the Fourier shell correlation (FSC) for maps generated from both MEMMI and random ensembles, in comparison with the experimental cryo-EM map for both the filament types, to further assess their agreement. The MEMMI ensemble in both cases (Fig. 5, E and F) exhibited better correlation with the cryo-EM map compared to the random ensemble, suggesting that it captures more relevant structural information. We also report the comparison of local correlations and FSC between single conformations from both the MEMMI and random ensembles, and the ensembles themselves, demonstrating that an ensemble provides a more accurate representation of the cryo-EM data than any single conformation (fig. S7). These results underscore the enhanced accuracy achieved by fitting structural ensembles to the cryo-EM maps, indicating that using the MEMMI technique leads to the generation of ensembles with higher fidelity to experimental data. To evaluate the influence on our results of the segmentation cutoff used to trim the experimental density map, we conducted a subsequent simulation using a larger cutoff of 10 Å around the atomistic model. Comparing the FSC curves (fig. S8) for the 6- and 10-Å back-calculated density maps with the experimental maps, we confirm that the chosen cutoff does not affect the reported data. Furthermore, an essential feature of MEMMI is its capability to estimate the error in the experimental density map. The average relative error per Gaussian data point was determined to be 0.2 for both filaments (fig. S9). Here, the relative error represents the deviation of each Gaussian data point concerning the total overlap between all data Gaussian mixture model (GMM) and the specific component of the data GMM (see Eq. 1).

### Ordered behavior of solvent molecules

**Fig. 6.**
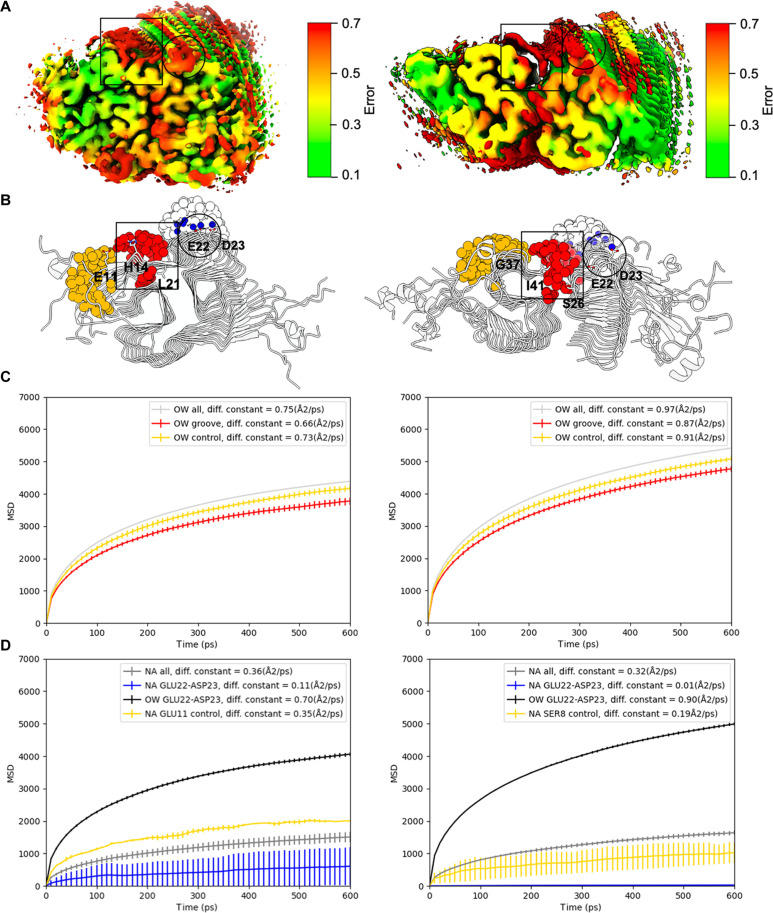
Analysis of the diffusion of water and ions near the surface of type I and type II filaments. (**A**) Lateral view of the projection in the Cryo-EM density maps of the relative error in the gmm data of type I (left) and type II (right) filaments; black squares indicate the high error volumes of interest. (**B**) Schematic representation of selected atoms for the calculation of the diffusion coefficient of water molecules and sodium ions. Red spheres denote oxygen atoms of water molecules moving within the groove defined by residues H14 and L21 for type I filament and by residues I41 and S26 for type II filament. Yellow spheres represent oxygen and sodium atoms of water molecules near residue E11 for type I filament and G37 and S8 for type II filament, used as controls. Blue spheres represent sodium atoms selected over residues E22 and D23. Transparent spheres highlight oxygen atoms of water molecules used as controls in comparison to sodium atoms. (**C** and **D**) Mean square displacement (MSD) analysis conducted on three replicas with the associated standard deviations are plotted over time for the water molecules (C) and sodium ions (D) experiments. Lines are color coded as in the schematic representation (B), with the exception of oxygen atom, shown in light gray, and all sodium ions, shown in gray, present in the simulation box and not shown in (B). In the legend, the diffusion constant for each selection is indicated.

The calculated correlation and error maps indicate discrepancies in specific regions of the experimental density map. These regions are not effectively captured by the MEMMI structural ensembles, resulting in relatively high error values (Fig. 6A). In both type I and type II filaments, these regions concern low-density volume data present over a groove formed around residues H14 and L21 by the filament structure (type I filaments), and at the interface around residues I41 and S26 of the two protofilaments (type II filaments). In addition, another volume with the same characteristics is observed over residues E22 and D23 in both filaments. A close-up examination of these regions reveals that the discrepancy is not associated with the density of the residues of the peptide. As previously highlighted, there are extra densities in the experimental density maps that are not utilized in modeling the atomic structure of the peptide. These additional densities may originate from solvent molecules with slower dynamics. To verify the hypothesis that solvation layer solvent molecules are trapped in these regions, exhibiting slower dynamics and therefore contributing to the experimental density map, we perform a solvent diffusion analysis for water and ions molecules during the MEMMI simulation (Fig. 6, B to D). Notably, for both filament types, the oxygen atoms of water molecules moving within the grooves exhibit a lower diffusion coefficient compared to the diffusion coefficient of the bulk water molecules in the simulation box and to water molecules near another point on the protein surface used as a control (around residues E11 for type I filament and G37, respectively; Fig. 6C). These findings support the conclusion that water molecules diffuse slower within these grooves, as a result of interactions with the protein, as found in different studies (*44*).

Furthermore, the densities observed in correspondence of residues E22 and D23 are linked to the capability of these two residues to coordinate positive ions (*17*). Consequently, we examine the diffusion of sodium ions. We find that the diffusion coefficient of the sodium ions moving over these two residues is lower than the diffusion coefficient of bulk sodium ions present in the simulated system (Fig. 6D). This observation suggests that sodium ions bind transiently residues E22 and D23 in both filament types. A comparison between the ordered molecules reported in the deposited PDB structures (7Q4B and 7Q4M) and those identified in our solvent diffusion analysis reveals a close match between the unknown nonprotein atoms in the PDB structures and the positions of the slowly diffusing sodium ions detected in the solvent diffusion analysis (fig. S10). In addition, some of the positions of the ordered oxygen atoms overlap. However, our analysis was primarily focused on identifying slow-diffusing water molecules rather than determining the positions of ordered water molecules. Notably, including the identified ordered water molecules and sodium ions in the back calculation of the cryo-EM map for their ensemble improves the local cross-correlation with the experimental map. Specifically, the correlation coefficient for the density map regions near the ordered molecules is calculated, and we observe that, for the type I fibril, the coefficient increases from 0.9354 to 0.9419, while for the type II fibril, it increases from 0.9063 to 0.9153, resulting in an about 1% increase in both cases, as one would expect from the fraction of atoms involved with respect to those of the whole system (fig. S11).

Overall, these findings demonstrate how water dynamics is influenced by the presence of the filaments. The characterization of this interplay provides an illustration of the complex influence that amyloid fibrils have on their environments.

## DISCUSSION

We have reported the conformational ensembles of two brain-derived amyloid filaments of Aβ42, which provide a description of their highly dynamical fuzzy coats. The analysis of these structural ensembles has enabled us to illustrate how the fuzzy coats interact with the cross-β cores of the filaments, thus influencing their accessibility to the environment and in turn the solubility of the filaments themselves.

Since the Bayesian approach adopted in the MEMMI method used here enables the determination of conformational ensembles consistent with the cryo-EM density maps while also estimating the errors resulting from the procedure, we have been able to characterize the slowing down of the diffusion of water molecules and sodium ions near the surface of the amyloid filaments.

On the basis of these results, we suggest that metainference approach described here will help analyze cryo-EM maps for the characterization of the properties of heterogeneous macromolecular systems.

## METHODS

### The MEMMI method

MEMMI (*37*) is a Bayesian method that models statistical ensembles of biomolecules by combining cryo-EM experimental data and metadynamics molecular dynamics to infer an ensemble of structures that maximally agree with the cryo-EM data while simultaneously inferring various sources of errors (e.g., data error, forward model error, and limited number of replicas able to model the heterogeneity of the system) (*38*). In MEMMI, the configurations of the structural ensemble and the different types of error are sampled from the following hybrid energy function

$$E_{\text{MEMMI}}\left(X,\sigma \right)=E_{\text{MD}}\left(X\right)+\frac{k_{B}T}{2}\sum_{r,i}^{N_{R},N_{D}}\frac{{\left[ov_{\text{DD},i}-{\bar{\mathrm{ov}}}_{\text{MD},i}\right]}^{2}}{{\left(\sigma_{r,i}^{B}\right)}^{2}+{\left(\sigma_{i}^{\text{SEM}}\right)}^{2}}+E_{\sigma }+\sum_{r=1}^{N_{R}}V_{\text{PB}}\left[s\left(X_{r}\right),t\right]$$ (1)

where $\phi_{D}\left(x\right)$ is data GMM conversion of the cryo-EM voxel map data to consisting of $N_{D}$ Gaussian components; $\phi_{M}\;\left(x\right)$ is the model GMM, a GMM conversion of the molecular dynamics models; ${\mathrm{ov}}_{\text{MD},i}$ is the overlap function quantifying the agreement between models generated by molecular dynamics (MD) and the data GMM that is calculated by the following overlap function ${\mathrm{ov}}_{\text{MD},i}$; $N_{R}$ is the number of replicas of the system, dealing with the heterogeneity of the system; ${\bar{\mathrm{ov}}}_{\mathrm{MD},i}$ is the overlap between model GMM and data GMM that is estimated over the ensemble of replicas as an average overlap per GMM component ${\bar{\mathrm{ov}}}_{\text{MD},i}$; ${\mathrm{ov}}_{\text{DD},i}$ is the data GMM self-overlap per datapoint; **X** is a vector representing the atomic coordinates of the full ensemble, consisting of individual replicas $X_{r}$; $\sigma_{i}^{\text{SEM}}$ is the error incurred by the limited number of replicas in the ensemble per datapoint; $\sigma_{r,i}^{B}$ is the error due to random and systematic errors in the prior, forward model, and experiment per datapoint and replica; $E_{\sigma }$ is the error energy associated with the error $\sigma =\left(\sigma^{B},\sigma^{\text{SEM}}\right)$ [see *40*)]; $E_{\text{MD}}$ is the force field; s(X) is the CV set (tables S1 and S2); and $V_{\text{PB}}$ is the time-dependent biasing potential acting on a set of $N_{\text{CV}}$ CVs (*45*). MEMMI samples the space of conformations $X_{r}$ by multi-replica molecular dynamics simulations, while the error parameters for each datapoint $\sigma_{r,i}^{B}$ are sampled by a Monte Carlo sampling scheme at each time step.

### Initial filament structure

We build the initial structure of the full length filament by starting from the PDB databank deposited type I (PDB: 7Q4B) and type II (PDB: 7Q4M) Aβ filament structures, which only contains the cross-β core of the filament and a five chains stacked on top of each other forming a β sheet, followed by extending the filament to a 12 chain stack by using Chimera (*46*) and the symmetry of each cross-β core (helical C1 and C2 symmetry, respectively) to avoid finite size effects in the generated structural ensemble. Then, by using RosettaFold (*47*, *48*), we model each the full-length polymorph filament by using each 12-stack cross-β core as a template and by providing the Aβ sequence. The cryo-EM density maps used as data in MEMMI are EMD-13800 and EMD-13809.

### Molecular dynamics setup and equilibration

For each full-length polymorph structure (type I and type II), we continue by creating a 11.99–by–7.40–by–9.51 nm and 9.30–by–13.68–by–9.56 nm simulation box, solvating with 22,334 and 35,090 water molecules, respectively, and neutralizing the net charge by adding 72 Na^+^ ions. We use the AMBER99SB-ILDN (*49*) force field and TIP3P (*50*) water models. We continued with an energy minimization followed by a 500-ps equilibration under conditions of constant number of particles (N), pressure (P), and temperature (T) (NPT) at a temperature of 310 K and pressure of 1 atm, followed by an additional 2-ns equilibration under conditions of constant number of particles (N), pressure (V), and temperature (T) (NVT) at 310 K. The molecular dynamics parameters are the same used previously (*30*, *31*).

### MEMMI simulations

We first fitted the obtained atomistic model to the respective experimental maps using Chimera. As reported in (*31*), at longer distances from the sample, the density maps contain a weak and low information content signal. To remove the low-resolution density map, we segmented the experimental maps EMD-13800 and EMD-13809 within 6 Å around the atomistic model obtaining a trimmed experimental map. We then express the trimmed experimental voxel maps data as a data GMM containing 22,492 and 22,496 Gaussians in total, resulting in a 0.878 and 0.885 correlation to the trimmed maps using the gmmconvert utility (*51*). We continued by extracting eight equally distant configurations throughout the 2-ns simulations from the previous NVT equilibration step and start MEMMI simulations, consisting of eight replicas, resulting in an aggregate runtime of 1 μs using PLUMED (*52*) (PLUMED.2.6.0-dev) and GROMACS (*53*) (GROMACS-2020.5). MEMMI is conducted in the NVT ensemble at 310 K using the same parameters as in the equilibration step. Configurations are saved every 10 ps for post-processing. The cryo-EM restraint is updated every two steps using neighbor lists to compute the overlaps between model and data GMMs, with a neighbor list cutoff of 0.01 and update frequency stride of 100 steps. The CVs used for biasing in the simulations are shown in tables S1 and S2, and the biasing scheme is PBMetaD (*45*) with the well-tempered (*54*) and multiple-walker (*55*) protocols. The hill height is set to 0.3 kJ/mol, with a deposition frequency of 200 steps and an adaptive Gaussians diffusion scheme (*51*). The biasing CVs correspond to degrees of freedom of the left hand-side N-terminal. The respective degrees of freedom of the right hand-side N-terminal do not feel a metadynamics potential. As a post-processing step, the initial 25 ns of each replica was excluded as equilibration, followed by the generation of the final structural ensemble by resampling the generated configurations based on the converged unbiasing weights. The converged unbiasing weights are obtained by following a previously reported procedure (*37*). To establish convergence, we calculated the CVs as a function of time (fig. S1), their time-dependent free energy profiles (fig. S2), and the block averaging analyses for each CV (fig. S3).

To evaluate the influence of the segmentation cutoff on our results, we performed a subsequent simulation using a larger cutoff of 10 Å around the atomistic model. Subsequently, we transformed the data from the 10-Å trimmed experimental voxel maps data into a data GMM. This yielded correlations of 0.871 and 0.876 with the trimmed maps. We followed the same protocol and molecular dynamics parameters as outlined earlier, resulting in a cumulative runtime of 0.6 μs.

### Contact map, SASA, and DSSP analysis

All the structural analysis is performed only on the two central chains of the fibrils to avoid the finite size effects on the outermost chains. To calculate the contact map, perform SASA analysis, and analyze the secondary structure population Dictionary of Secondary Structure of Proteins (DSSP) the mdtraj (63) Python program was used. In the contact map analysis, a contact is considered to occur when the distance between the center of mass of two residues is less than 1 nm. For the classification of the type of noncovalent interaction between the N-terminal residues and the cross-β core residues the getcontact application (https://getcontacts.github.io/) were used. The SASA analysis used the per-residue Shrake-Rupley function, with a selected probe radius of 0.34.

### Local correlation, FSC curves, and relative error

We initially generate a structural ensemble of 100 conformations with random N-terminal structures using the Model Loops tool in Chimera. We then back-calculate electron density maps for the 100 structures in both the MEMMI resampled ensemble and the random ensemble using EMAN2 (*56*). For molecular visualizations and calculating the local correlation between the cryo-EM maps generated by the MEMMI and random ensembles and the experimental cryo-EM map, we use Chimera. The FSC curves between the back-calculated density maps and the experimental cryo-EM map are generated using EMAN2. This procedure was carried out in three replicates for both MEMMI and random ensembles. The mean for each FSC curve point and the respective SD were then calculated. For the calculation of the relative error in the gmm data, the gmmconvert utility and Chimera are used. The detailed commands used are available in the Jupyter notebook on Zenodo (https://doi.org/10.5281/zenodo.14354380).

### Solvent diffusion analysis for water molecules and sodium ions

To calculate the diffusion coefficient of water molecules or sodium ions, the mdanalysis toolkit was used (*57*). For each case, a cylinder centered at the middle chain along the filaments at residues E11 or H14/L21 or E22/D23 for type I filaments and G37 or I41/S26 or E22-D23 for type II filaments. Around these centers, the radius of each cylinder was set to 1 nm, and the height of the cylinder was chosen as 3.6 nm so that it covers the axis length of the filaments. Within these cylinders, the mean square deviation (MSD) of the oxygen atoms of water molecules or of the sodium ion was calculated for each of the three trajectories, and average and SDs were obtained. From the averaged MSD, we used the Einstein equation to calculate the diffusion constant.

## References

[1] M. Prince, A. Wimo, M. Guerchet, G.-C. Ali, Y.-T. Wu, M. Prina, World Alzheimer report 2015. The global impact of dementia: An analysis of prevalence, incidence, cost and trends. Alzheimer’s Disease International (2015).

[2] A. Wimo, K. Seeher, R. Cataldi, E. Cyhlarova, J. L. Dielemann, O. Frisell, M. Guerchet, L. Jönsson, A. K. Malaha, E. Nichols, P. Pedroza, M. Prince, M. Knapp, T. Dua, The worldwide costs of dementia in 2019. Alzheimers Dement. 19, 2865–2873 (2023). 36617519 10.1002/alz.12901 PMC10842637

[3] D. J. Selkoe, J. Hardy, The amyloid hypothesis of Alzheimer’s disease at 25 years. EMBO Mol. Med. 8, 595–608 (2016). 27025652 10.15252/emmm.201606210 PMC4888851

[4] C. R. Jack Jr., D. A. Bennett, K. Blennow, M. C. Carrillo, B. Dunn, S. B. Haeberlein, D. M. Holtzman, W. Jagust, F. Jessen, J. Karlawish, E. Liu, J. L. Molinuevo, T. Montine, C. Phelps, K. P. Rankin, C. C. Rowe, P. Scheltens, E. Siemers, H. M. Synder, R. Sperling, Contributors, NIA-AA research framework: Toward a biological definition of Alzheimer’s disease. Alzheimers Dement. 14, 535–562 (2018). 29653606 10.1016/j.jalz.2018.02.018 PMC5958625

[5] H. Hampel, J. Hardy, K. Blennow, C. Chen, G. Perry, S. H. Kim, V. L. Villemagne, P. Aisen, M. Vendruscolo, T. Iwatsubo, C. L. Masters, M. Cho, L. Lannfelt, J. L. Cummings, A. Vergallo, The amyloid-β pathway in Alzheimer’s disease. Mol. Psychiatry 26, 5481–5503 (2021). 34456336 10.1038/s41380-021-01249-0 PMC8758495

[6] T. P. J. Knowles, M. Vendruscolo, C. M. Dobson, The amyloid state and its association with protein misfolding diseases. Nat. Rev. Mol. Cell Biol. 15, 384–396 (2014). 24854788 10.1038/nrm3810

[7] S. I. A. Cohen, S. Linse, L. M. Luheshi, E. Hellstrand, D. A. White, L. Rajah, D. E. Otzen, M. Vendruscolo, C. M. Dobson, T. P. J. Knowles, Proliferation of amyloid-β42 aggregates occurs through a secondary nucleation mechanism. Proc. Natl. Acad. Sci. U.S.A. 110, 9758–9763 (2013). 23703910 10.1073/pnas.1218402110 PMC3683769

[8] D. Eisenberg, M. Jucker, The amyloid state of proteins in human diseases. Cell 148, 1188–1203 (2012). 22424229 10.1016/j.cell.2012.02.022 PMC3353745

[9] Y. Shi, W. Zhang, Y. Yang, A. G. Murzin, B. Falcon, A. Kotecha, M. van Beers, A. Tarutani, F. Kametani, H. J. Garringer, R. Vidal, G. I. Hallinan, T. Lashley, Y. Saito, S. Marumaya, M. Yoshida, H. Tanaka, A. Kakita, T. Ikeuchi, A. C. Robinson, D. M. A. Mann, G. G. Kovacs, T. Revesz, B. Ghetti, M. Hasegawa, M. Goedert, S. H. W. Scheres, Structure-based classification of tauopathies. Nature 598, 359–363 (2021). 34588692 10.1038/s41586-021-03911-7 PMC7611841

[10] A. T. Petkova, R. D. Leapman, Z. Guo, W.-M. Yau, M. P. Mattson, R. Tycko, Self-propagating, molecular-level polymorphism in Alzheimer’s ß-amyloid fibrils. Science 307, 262–265 (2005). 15653506 10.1126/science.1105850

[11] C. Sachse, M. Fändrich, N. Grigorieff, Paired β-sheet structure of an Aβ (1-40) amyloid fibril revealed by electron microscopy. Proc. Natl. Acad. Sci. U.S.A. 105, 7462–7466 (2008). 18483195 10.1073/pnas.0712290105 PMC2396686

[12] M. T. Colvin, R. Silvers, Q. Z. Ni, T. V. Can, I. Sergeyev, M. Rosay, K. J. Donovan, B. Michael, J. Wall, S. Linse, R. G. Griffin, Atomic resolution structure of monomorphic Aβ42 amyloid fibrils. J. Am. Chem. Soc. 138, 9663–9674 (2016). 27355699 10.1021/jacs.6b05129 PMC5389415

[13] L. Gremer, D. Schölzel, C. Schenk, E. Reinartz, J. Labahn, R. B. G. Ravelli, M. Tusche, C. Lopez-Iglesias, W. Hoyer, H. Heise, D. Willbold, G. F. Schröder, Fibril structure of amyloid-β (1–42) by cryo–electron microscopy. Science 358, 116–119 (2017). 28882996 10.1126/science.aao2825 PMC6080689

[14] M. A. Wälti, F. Ravotti, H. Arai, C. G. Glabe, J. S. Wall, A. Böckmann, P. Güntert, B. H. Meier, R. Riek, Atomic-resolution structure of a disease-relevant Aβ (1–42) amyloid fibril. Proc. Natl. Acad. Sci. U.S.A. 113, E4976–E4984 (2016). 27469165 10.1073/pnas.1600749113 PMC5003276

[15] J.-X. Lu, W. Qiang, W.-M. Yau, C. D. Schwieters, S. C. Meredith, R. Tycko, Molecular structure of β-amyloid fibrils in Alzheimer’s disease brain tissue. Cell 154, 1257–1268 (2013). 24034249 10.1016/j.cell.2013.08.035 PMC3814033

[16] M. Kollmer, W. Close, L. Funk, J. Rasmussen, A. Bsoul, A. Schierhorn, M. Schmidt, C. J. Sigurdson, M. Jucker, M. Fändrich, Cryo-EM structure and polymorphism of Aβ amyloid fibrils purified from Alzheimer’s brain tissue. Nat. Commun. 10, 4760 (2019). 31664019 10.1038/s41467-019-12683-8 PMC6820800

[17] Y. Yang, D. Arseni, W. Zhang, M. Huang, S. Lövestam, M. Schweighauser, A. Kotecha, A. G. Murzin, S. Y. Peak-Chew, J. Macdonald, I. Lavenir, H. J. Garringer, E. Gelpi, K. L. Newell, G. G. Kovacs, R. Vidal, B. Ghetti, B. Ryskeldi-Falcon, S. H. W. Scheres, M. Goedert, Cryo-EM structures of amyloid-β 42 filaments from human brains. Science 375, 167–172 (2022). 35025654 10.1126/science.abm7285 PMC7612234

[18] P. Tompa, Structural disorder in amyloid fibrils: Its implication in dynamic interactions of proteins. FEBS J. 276, 5406–5415 (2009). 19712107 10.1111/j.1742-4658.2009.07250.x

[19] S. M. Ulamec, D. J. Brockwell, S. E. Radford, Looking beyond the core: The role of flanking regions in the aggregation of amyloidogenic peptides and proteins. Front. Neurosci. 14, 611285 (2020). 33335475 10.3389/fnins.2020.611285 PMC7736610

[20] S. Wegmann, I. D. Medalsy, E. Mandelkow, D. J. Müller, The fuzzy coat of pathological human tau fibrils is a two-layered polyelectrolyte brush. Proc. Natl. Acad. Sci. U.S.A. 110, E313–E321 (2013). 23269837 10.1073/pnas.1212100110 PMC3557036

[21] A. A. Bhopatkar, R. Kayed, Flanking regions, amyloid cores, and polymorphism: The potential interplay underlying structural diversity. J. Biol. Chem. 299, 105122 (2023). 37536631 10.1016/j.jbc.2023.105122 PMC10482755

[22] M. Bonomi, R. Pellarin, M. Vendruscolo, Simultaneous determination of protein structure and dynamics using cryo-electron microscopy. Biophys. J. 114, 1604–1613 (2018). 29642030 10.1016/j.bpj.2018.02.028 PMC5954442

[23] M. Bonomi, M. Vendruscolo, Determination of protein structural ensembles using cryo-electron microscopy. Curr. Opin. Struct. Biol. 56, 37–45 (2019). 30502729 10.1016/j.sbi.2018.10.006

[24] S. Matsumoto, S. Ishida, M. Araki, T. Kato, K. Terayama, Y. Okuno, Extraction of protein dynamics information from cryo-em maps using deep learning. Nat. Mach. Intell. 3, 153–160 (2021).

[25] L. Eshun-Wilson, R. Zhang, D. Portran, M. V. Nachury, D. B. Toso, T. Löhr, M. Vendruscolo, M. Bonomi, J. S. Fraser, E. Nogales, Effects of α-tubulin acetylation on microtubule structure and stability. Proc. Natl. Acad. Sci. U.S.A. 116, 10366–10371 (2019). 31072936 10.1073/pnas.1900441116 PMC6535015

[26] J. Giraldo-Barreto, S. Ortiz, E. H. Thiede, K. Palacio-Rodriguez, B. Carpenter, A. H. Barnett, P. Cossio, A Bayesian approach to extracting free-energy profiles from cryo-electron microscopy experiments. Sci. Rep. 11, 13657 (2021). 34211017 10.1038/s41598-021-92621-1 PMC8249403

[27] M. Chen, S. J. Ludtke, Deep learning-based mixed-dimensional gaussian mixture model for characterizing variability in cryo-em. Nat. Methods 18, 930–936 (2021). 34326541 10.1038/s41592-021-01220-5 PMC8363932

[28] L. F. Kinman, B. M. Powell, E. D. Zhong, B. Berger, J. H. Davis, Uncovering structural ensembles from single-particle cryo-em data using cryoDRGN. Nat. Protoc. 18, 319–339 (2023). 36376590 10.1038/s41596-022-00763-x PMC10049411

[29] D. Herreros, R. R. Lederman, J. M. Krieger, A. Jiménez-Moreno, M. Martínez, D. Myška, D. Strelak, J. Filipovic, C. O. S. Sorzano, J. M. Carazo, Estimating conformational landscapes from Cryo-EM particles by 3D zernike polynomials. Nat. Commun. 14, 154 (2023). 36631472 10.1038/s41467-023-35791-y PMC9832421

[30] H. Mikolajek, M. Weckener, Z. F. Brotzakis, J. Huo, E. V. Dalietou, A. Le Bas, P. Sormanni, P. J. Harrison, P. N. Ward, S. Truong, L. Moynie, D. K. Clare, M. Dumoux, J. Dormon, C. Norman, N. Hussain, V. Vogirala, R. J. Owens, M. Vendruscolo, J. H. Naismith, Correlation between the binding affinity and the conformational entropy of nanobody SARS-CoV-2 spike protein complexes. Proc. Natl. Acad. Sci. U.S.A. 119, e2205412119 (2022). 35858383 10.1073/pnas.2205412119 PMC9351521

[31] Z. F. Brotzakis, P. R. Lindstedt, R. J. Taylor, D. J. Rinauro, N. C. T. Gallagher, G. J. L. Bernardes, M. Vendruscolo, A structural ensemble of a tau-microtubule complex reveals regulatory tau phosphorylation and acetylation mechanisms. ACS Cent. Sci. 7, 1986–1995 (2021). 34963892 10.1021/acscentsci.1c00585 PMC8704032

[32] Y. Hori, T. Hashimoto, Y. Wakutani, K. Urakami, K. Nakashima, M. M. Condron, S. Tsubuki, T. C. Saido, D. B. Teplow, T. Iwatsubo, The tottori (D7N) and english (H6R) familial Alzheimer disease mutations accelerate Aβ fibril formation without increasing protofibril formation. J. Biol. Chem. 282, 4916–4923 (2007). 17170111 10.1074/jbc.M608220200

[33] L. M. Luheshi, G. G. Tartaglia, A.-C. Brorsson, A. P. Pawar, I. E. Watson, F. Chiti, M. Vendruscolo, D. A. Lomas, C. M. Dobson, D. C. Crowther, Systematic in vivo analysis of the intrinsic determinants of amyloid β pathogenicity. PLOS Biol. 5, e290 (2007). 17973577 10.1371/journal.pbio.0050290 PMC2043051

[34] W.-T. Chen, C.-J. Hong, Y.-T. Lin, W.-H. Chang, H.-T. Huang, J.-Y. Liao, Y.-J. Chang, Y.-F. Hsieh, C.-Y. Cheng, H.-C. Liu, Y.-R. Chen, I. H. Cheng, Amyloid-beta (Aβ) d7h mutation increases oligomeric Aβ42 and alters properties of Aβ-zinc/copper assemblies. PLOS ONE 7, e35807 (2012). 22558227 10.1371/journal.pone.0035807 PMC3340413

[35] J.-M. Shi, H.-Y. Li, H. Liu, L. Zhu, Y.-B. Guo, J. Pei, H. An, Y.-S. Li, S.-D. Li, Z.-Y. Zhang, Y. Zheng, N-terminal domain of amyloid-β impacts fibrillation and neurotoxicity. ACS Omega 7, 38847–38855 (2022). 36340079 10.1021/acsomega.2c04583 PMC9631750

[36] B. V. Foroutanpay, J. Kumar, S. G. Kang, N. Danaei, D. Westaway, V. L. Sim, S. Kar, The effects of N-terminal mutations on β-amyloid peptide aggregation and toxicity. Neuroscience 379, 177–188 (2018). 29572166 10.1016/j.neuroscience.2018.03.014

[37] Z. F. Brotzakis, T. Löhr, S. Truong, S. Hoff, M. Bonomi, M. Vendruscolo, Determination of the structure and dynamics of the fuzzy coat of an amyloid fibril of iapp using cryo-electron microscopy. Biochemistry 62, 2407–2416 (2023). 37477459 10.1021/acs.biochem.3c00010 PMC10433526

[38] A. Laio, M. Parrinello, Escaping free-energy minima. Proc. Natl. Acad. Sci. U.S.A. 99, 12562–12566 (2002). 12271136 10.1073/pnas.202427399 PMC130499

[39] G. Bussi, A. Laio, Using metadynamics to explore complex free-energy landscapes. Nat. Rev. Phys. 2, 200–212 (2020).

[40] V. N. Sivanandam, M. Jayaraman, C. L. Hoop, R. Kodali, R. Wetzel, P. C. A. van der Wel, The aggregation-enhancing huntingtin n-terminus is helical in amyloid fibrils. J. Am. Chem. Soc. 133, 4558–4566 (2011). 21381744 10.1021/ja110715f PMC3109494

[41] G. T. Heller, F. A. Aprile, T. C. T. Michaels, R. Limbocker, M. Perni, F. S. Ruggeri, B. Mannini, T. Löhr, M. Bonomi, C. Camilloni, A. De Simone, I. C. Felli, R. Pierattelli, T. P. J. Knowles, C. M. Dobson, M. Vendruscolo, Small-molecule sequestration of amyloid-β as a drug discovery strategy for Alzheimer’s disease. Sci. Adv. 6, eabb5924 (2020). 33148639 10.1126/sciadv.abb5924 PMC7673680

[42] P. Sormanni, F. A. Aprile, M. Vendruscolo, The CamSol method of rational design of protein mutants with enhanced solubility. J. Mol. Biol. 427, 478–490 (2015). 25451785 10.1016/j.jmb.2014.09.026

[43] M. Oeller, R. J. D. Kang, H. L. Bolt, A. L. Gomes dos Santos, A. L. Weinmann, A. Nikitidis, P. Zlatoidsky, W. Su, W. Czechtizky, L. De Maria, P. Sormanni, M. Vendruscolo, Sequence-based prediction of the intrinsic solubility of peptides containing non-natural amino acids. Nat. Commun. 14, 7475 (2023). 37978172 10.1038/s41467-023-42940-w PMC10656490

[44] Z. F. Brotzakis, C. C. M. Groot, W. H. Brandeburgo, H. J. Bakker, P. G. Bolhuis, Dynamics of hydration water around native and misfolded α-lactalbumin. J. Phys. Chem. B 120, 4756–4766 (2016). 27137845 10.1021/acs.jpcb.6b02592

[45] J. Pfaendtner, M. Bonomi, Efficient sampling of high-dimensional free-energy landscapes with parallel bias metadynamics. J. Chem. Theory Comput. 11, 5062–5067 (2015). 26574304 10.1021/acs.jctc.5b00846

[46] E. F. Pettersen, T. D. Goddard, C. C. Huang, G. S. Couch, D. M. Greenblatt, E. C. Meng, T. E. Ferrin, UCSF chimera—a visualization system for exploratory research and analysis. J. Comput. Chem. 25, 1605–1612 (2004). 15264254 10.1002/jcc.20084

[47] M. Baek, F. DiMaio, I. Anishchenko, J. Dauparas, S. Ovchinnikov, G. R. Lee, J. Wang, Q. Cong, L. N. Kinch, R. D. Schaeffer, C. Millán, H. Park, C. Adams, C. R. Glassman, A. Degiovanni, J. H. Pereira, A. V. Rodrigues, A. A. Van Dijk, A. C. Ebrecht, D. J. Opperman, T. Sagmeister, C. Buhlheller, T. Pavkov-Keller, M. K. Rathinaswamy, U. Dalwadi, C. K. Yip, J. E. Burke, K. C. Garcia, N. V. Grishin, P. D. Adams, R. J. Read, D. Baker, Accurate prediction of protein structures and interactions using a three-track neural network. Science 373, 871–876 (2021). 34282049 10.1126/science.abj8754 PMC7612213

[48] Y. Song, F. DiMaio, R. Y.-R. Wang, D. Kim, C. Miles, T. Brunette, J. Thompson, D. Baker, High-resolution comparative modeling with rosettacm. Structure 21, 1735–1742 (2013). 24035711 10.1016/j.str.2013.08.005 PMC3811137

[49] K. Lindorff-Larsen, S. Piana, K. Palmo, P. Maragakis, J. L. Klepeis, R. O. Dror, D. E. Shaw, Improved side-chain torsion potentials for the Amber ff99sb protein force field. Proteins 78, 1950–1958 (2010). 20408171 10.1002/prot.22711 PMC2970904

[50] W. L. Jorgensen, J. Chandrasekhar, J. D. Madura, R. W. Impey, M. L. Klein, Comparison of simple potential functions for simulating liquid water. J. Chem. Phys. 79, 926–935 (1983).

[51] D. Branduardi, G. Bussi, M. Parrinello, Metadynamics with adaptive gaussians. J. Chem. Theory Comput. 8, 2247–2254 (2012). 26588957 10.1021/ct3002464

[52] M. Bonomi, G. Bussi, C. Camilloni, G. A. Tribello, P. Banáš, A. Barducci, M. Bernetti, P. G. Bolhuis, S. Bottaro, D. Branduardi, Promoting transparency and reproducibility in enhanced molecular simulations. Nat. Methods 16, 670–673 (2019). 31363226 10.1038/s41592-019-0506-8

[53] M. J. Abraham, T. Murtola, R. Schulz, S. Páll, J. C. Smith, B. Hess, E. Lindahl, GROMACS: High performance molecular simulations through multi-level parallelism from laptops to supercomputers. SoftwareX 1-2, 19–25 (2015).

[54] A. Barducci, G. Bussi, M. Parrinello, Well-tempered metadynamics: A smoothly converging and tunable free-energy method. Phys. Rev. Lett. 100, 020603 (2008). 18232845 10.1103/PhysRevLett.100.020603

[55] P. Raiteri, A. Laio, F. L. Gervasio, C. Micheletti, M. Parrinello, Efficient reconstruction of complex free energy landscapes by multiple walkers metadynamics. J. Phys. Chem. B 110, 3533–3539 (2006). 16494409 10.1021/jp054359r

[56] G. Tang, L. Peng, P. R. Baldwin, D. S. Mann, W. Jiang, I. Rees, S. J. Ludtke, EMAN2: An extensible image processing suite for electron microscopy. J. Struct. Biol. 157, 38–46 (2007). 16859925 10.1016/j.jsb.2006.05.009

[57] R. J. Gowers, M. Linke, J. Barnoud, T. J. Reddy, M. N. Melo, S. L. Seyler, J. Domanski, D. L. Dotson, S. Buchoux, I. M. Kenney, in *Proceedings of the 15th Python in Science Conference* (SciPy, 2016), vol. 98, pp. 105.

