# Supplementary material for: Transient interactions between the fuzzy coat and the cross-β core of brain-derived Aβ42 filaments
Source: Sci Adv. 2025 Jan 15;11(3):eadr7008. doi: 10.1126/sciadv.adr7008 (PMC11734738; doi:10.1126/sciadv.adr7008)
Supplement: Supplementary file 1 — Tables S1 and S2 Figs. S1 to S11 [file sciadv.adr7008_sm.pdf]

Science Advances
AAAS

Supplementary Materials for

Transient interactions between the fuzzy coat and the cross-β core of brain-derived Aβ42 filaments

Maria Milanesi *et al.*

Corresponding author: Michele Vendruscolo, mv245@cam.ac.uk

Sci. Adv. **11**, eadr7008 (2025)
DOI: 10.1126/sciadv.adr7008

This PDF file includes:

Tables S1 and S2
Figs. S1 to S11

Description	Group
RESIDUES=1-8	tail0
RESIDUES=85-92	tail2
RESIDUES=169-176	tail4
RESIDUES=253-260	tail6
RESIDUES=337-344	tail8
RESIDUES=421-428	tail10
RESIDUES=505-512	tail12
RESIDUES=589-596	tail14
RESIDUES=673-680	tail16
RESIDUES=757-764	tail18
RESIDUES=841-848	tail20
RESIDUES=925-932	tail22
RESIDUES=18-21	epitope0
RESIDUES=102-105	epitope2
RESIDUES=186-189	epitope4
RESIDUES=270-273	epitope6
RESIDUES=354-357	epitope8
RESIDUES=438-441	epitope10
RESIDUES=522-525	epitope12
RESIDUES=606-609	epitope14
RESIDUES=690-693	epitope16
RESIDUES=774-777	epitope18
RESIDUES=858-861	epitope20
RESIDUES=942-945	epitope22

Table S1. List of residue groups used in the definition of the collective variables (CVs) used for biasing (**Table S2**).

Description	Plumed file notation	Manuscript Notation
Coordination tail0 and tail2	con0	#C0
Coordination tail0 and tail4	con1	#C1
Coordination tail4 and tail22	con2	#C2
Coordination tail20 and tail22	con3	#C3
Coordination tail2 and tail6	con4	#C4
Coordination tail6 and tail8	con5	#C5
Coordination tail8 and tail14	con6	#C6
Coordination tail10 and tail14	con7	#C7
Coordination tail10 and tail12	con8	#C8
Coordination tail12 and tail16	con9	#C9
Coordination tail16 and tail18	con10	#C10
Distance between the center of mass tail0 and epitope0	dcm_0	#d1
Distance between the center of mass tail2 and epitope2	dcm_2	#d2
Distance between the center of mass tail4 and epitope4	dcm_4	#d3
Distance between the center of mass tail6 and epitope6	dcm_6	#d4
Distance between the center of mass tail8 and epitope8	dcm_8	#d5
Distance between the center of mass tail10 and epitope10	dcm_10	#d6
Distance between the center of mass tail12 and epitope12	dcm_12	#d7
Distance between the center of mass tail14 and epitope14	dcm_14	#d8
Distance between the center of mass tail16 and epitope16	dcm_16	#d9
Distance between the center of mass tail18 and epitope18	dcm_18	#d10
Distance between the center of mass tail20 and epitope20	dcm_20	#d11
Distance between the center of mass tail22 and epitope22	dcm_22	#d12
alpha helical content tail0	alpha0	#ah1
alpha helical content tail2	alpha2	#ah2
alpha helical content tail4	alpha4	#ah3
alpha helical content tail6	alpha6	#ah4
alpha helical content tail8	alpha8	#ah5
alpha helical content tail10	alpha10	#ah6
alpha helical content tail12	alpha12	#ah7
alpha helical content tail14	alpha14	#ah8
alpha helical content tail16	alpha16	#ah9
alpha helical content tail18	alpha18	#ah10
alpha helical content tail20	alpha20	#ah11
alpha helical content tail22	alpha22	#ah12
parallel beta sheet content tail0	parabeta0	#bs1
parallel beta sheet content tail2	parabeta2	#bs2
parallel beta sheet content tail4	parabeta4	#bs3
parallel beta sheet content tail6	parabeta6	#bs4
parallel beta sheet content tail8	parabeta8	#bs5
parallel beta sheet content tail10	parabeta10	#bs6
parallel beta sheet content tail12	parabeta12	#bs7
parallel beta sheet content tail14	parabeta14	#bs8
parallel beta sheet content tail16	parabeta16	#bs9
parallel beta sheet content tail18	parabeta18	#bs10
parallel beta sheet content tail20	parabeta20	#bs11
parallel beta sheet content tail22	parabeta22	#bs12

Table S2. List of collective variables (CVs) used for biasing.

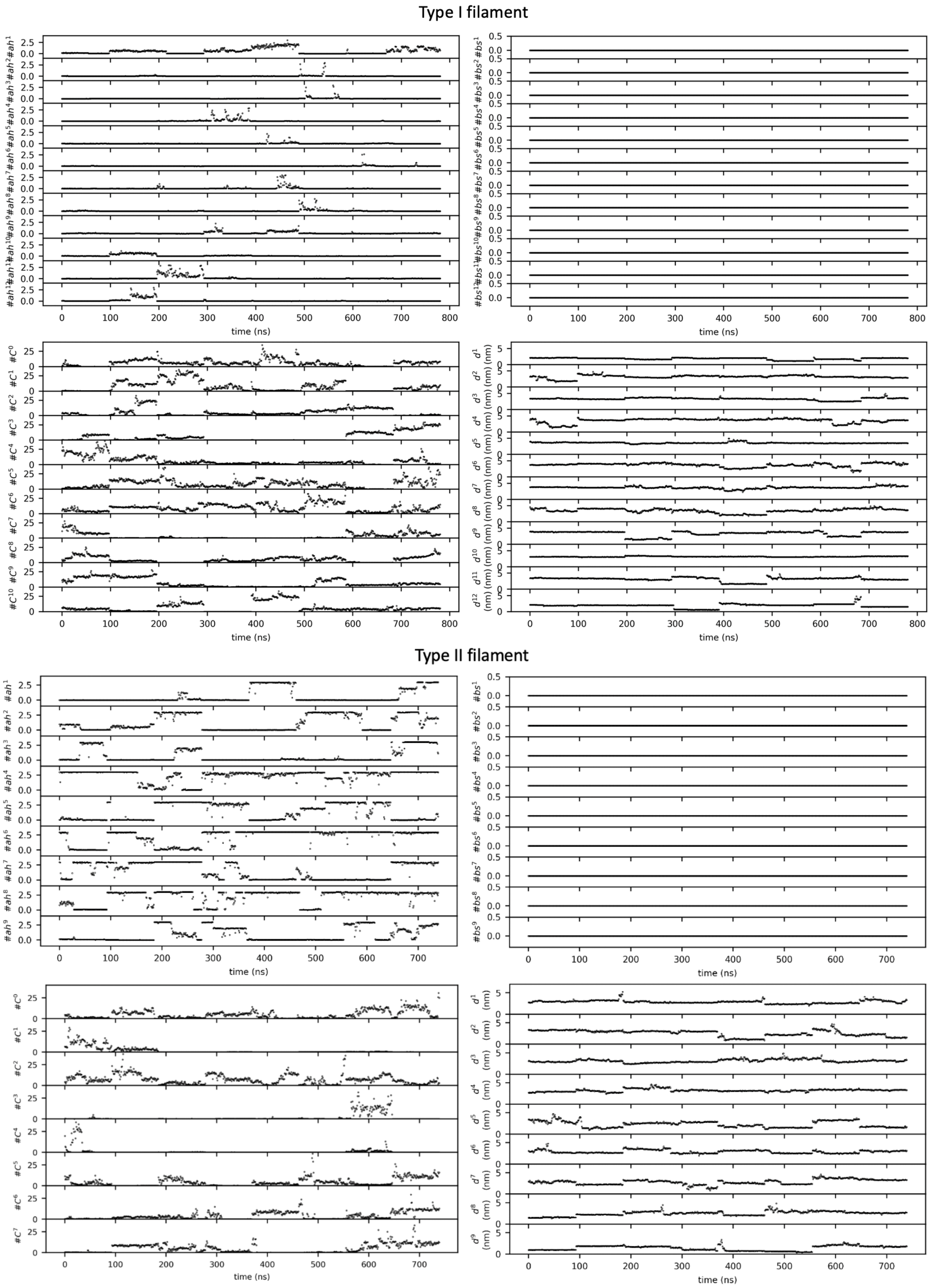

Figure S1. Assessment of the convergence of the MEMMI simulations. Time evolution profiles for all biased CVs for type I filament (up) and type II filament (down).

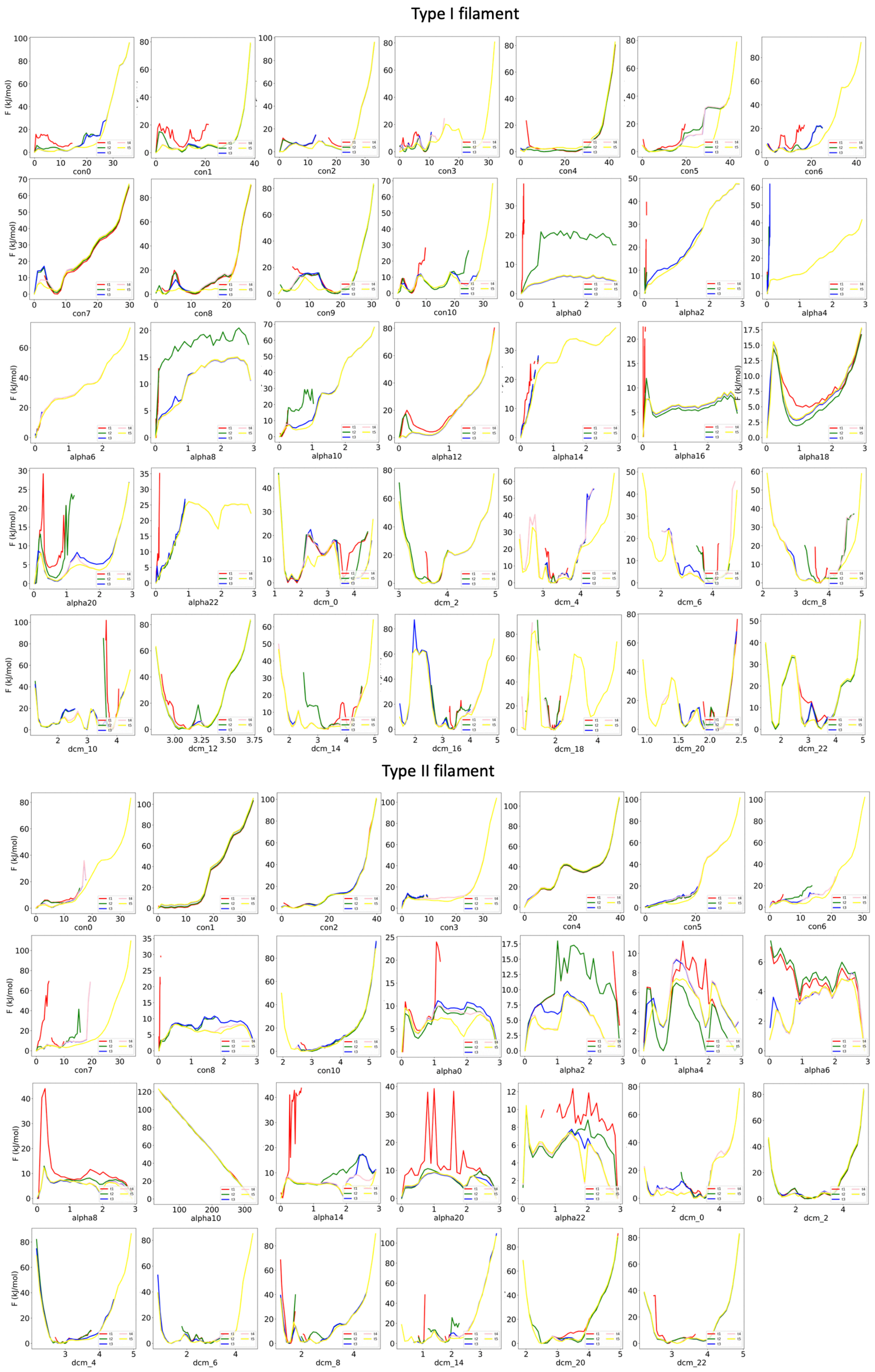

Figure S2. Assessment of the convergence of the MEMMI simulations. Free energy profiles for all biased CVs for subsequent 1 μs increments of simulated time.

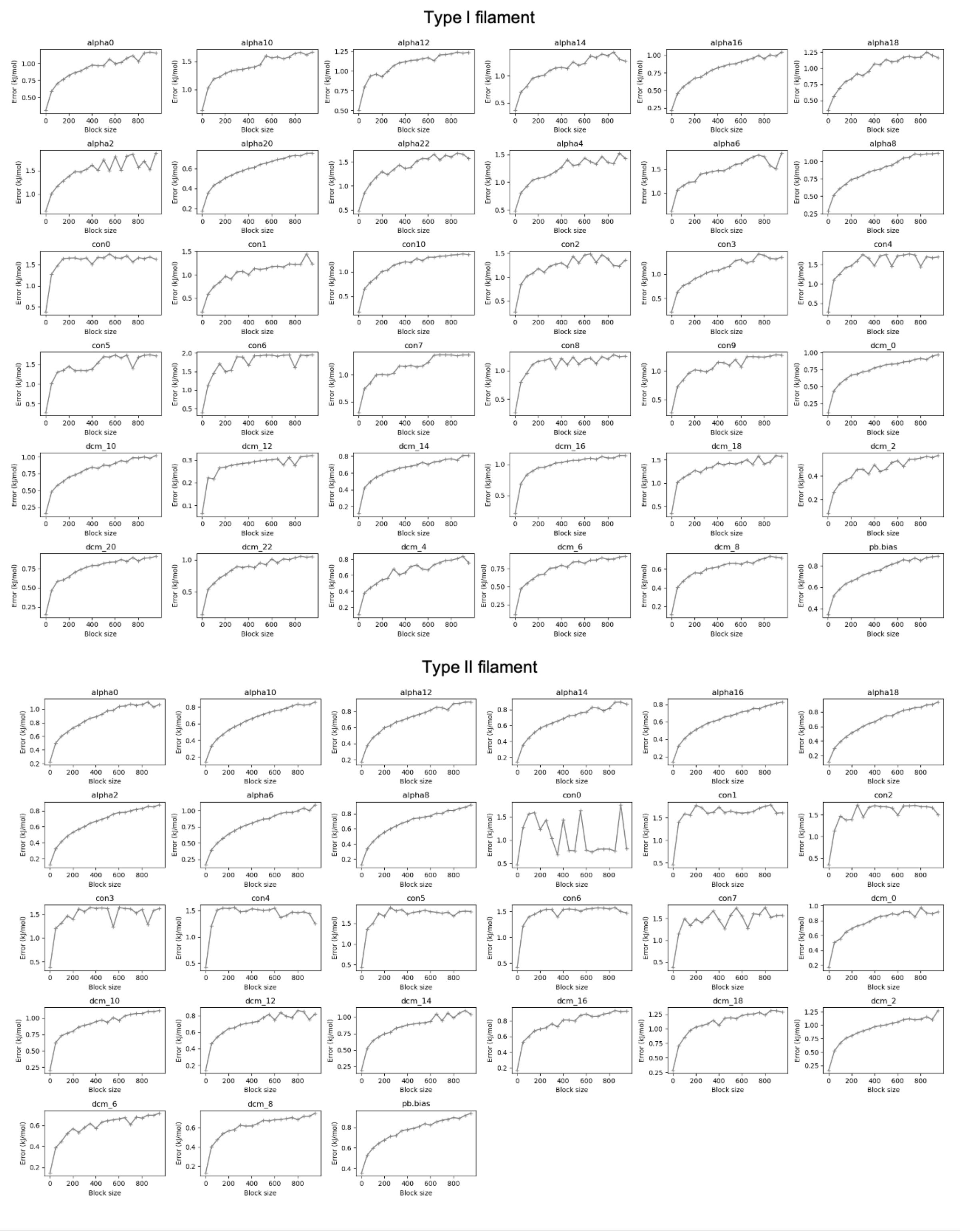

Figure S3. Assessment of the convergence of the MEMMI simulations via block analysis for each CV trace. Each plot illustrates the average error along the free-energy profile of each CVs as a function of the block length.

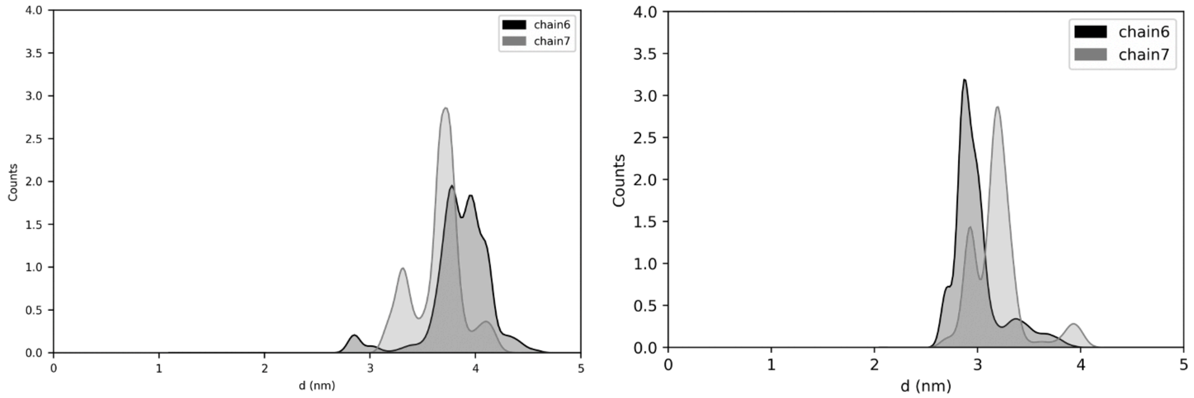

Figure S4. Distance distribution between the fuzzy coat and the cross-β core of type I and type II filaments. Distribution of the distance between the N-terminus (residues 1-9) and the cross-β core (residues 16-21) of the two central chains (chain 6 and chain 7) during the simulation. Type I filament (left panel) and type II filament (right panel).

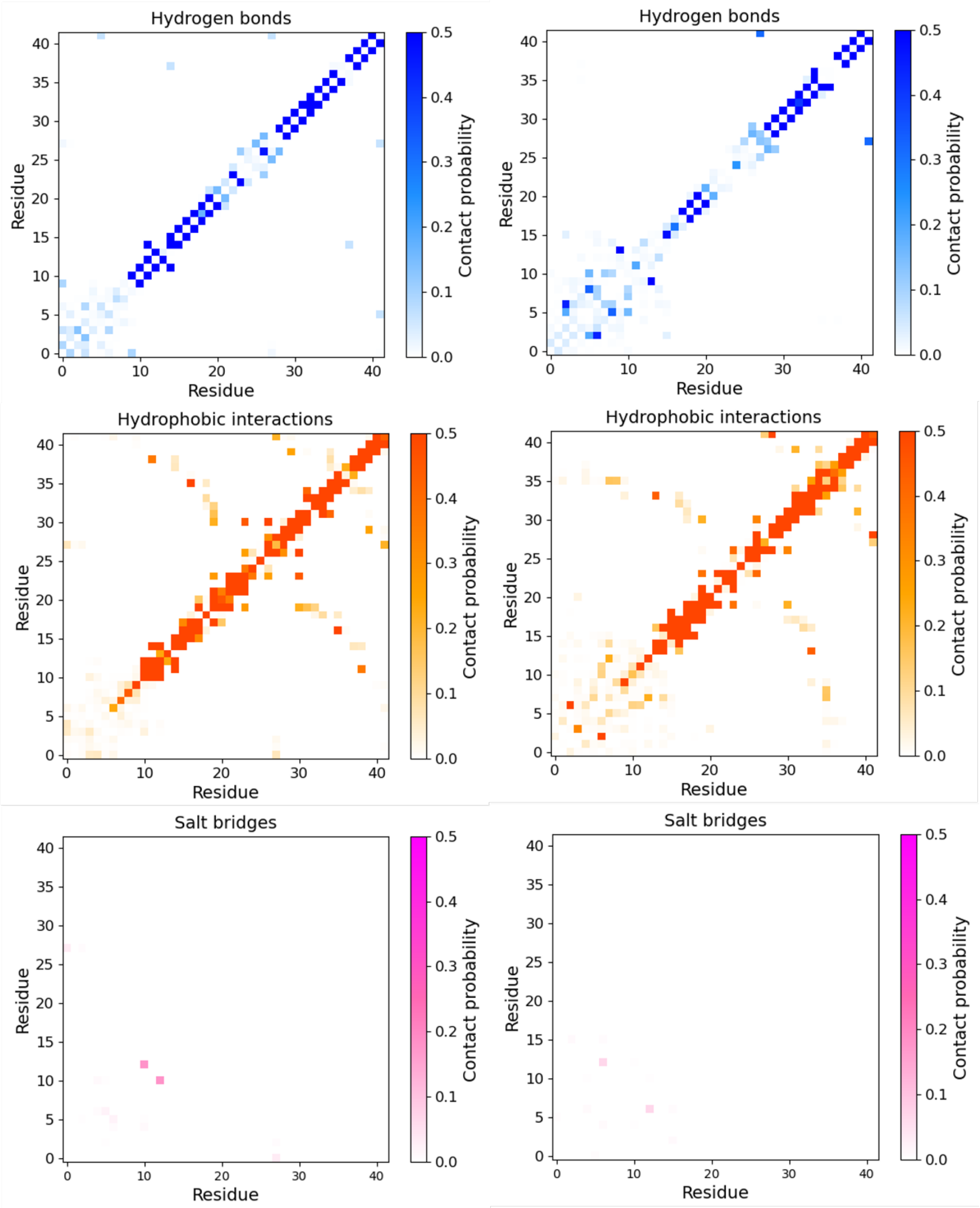

Figure S5. Contact maps subdivided by type of interaction. Contact maps corresponding to the structural ensembles of type I (left panels) and type II (right panels) filaments. The contact maps show the contact probability between residue pairs for each specific type of interaction, hydrogen bonds in blue (upper panels), hydrophobic interactions in orange (central panels) and salt bridges in magenta (lower panels).

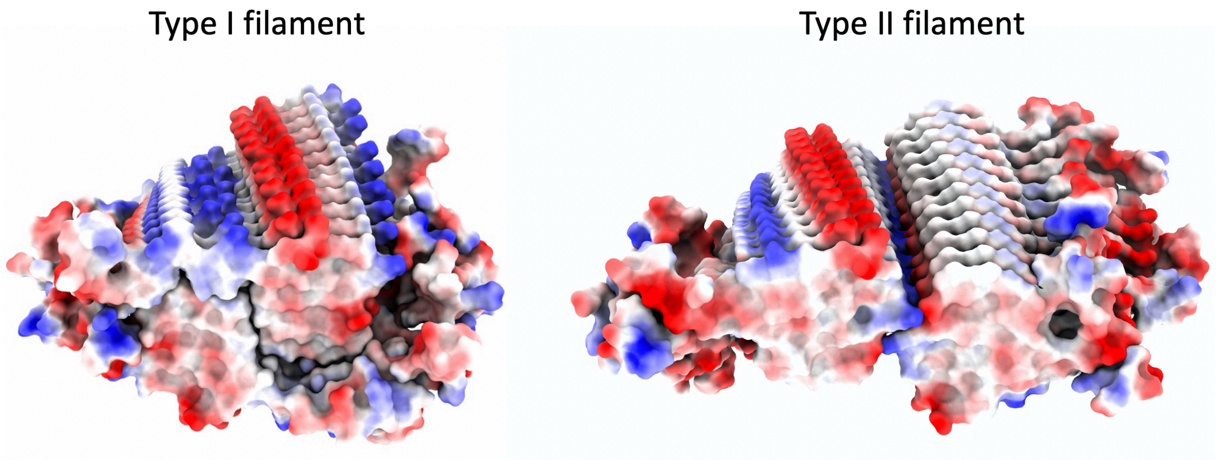

Figure S6. Surface electrostatic potential of type I and type II filaments. Most representative structure of the MEMMI structural ensemble of type I filament (left) and type II filament (right). Electrostatic potential maps are colored in blue to indicate positive potential and in red for negative potential.

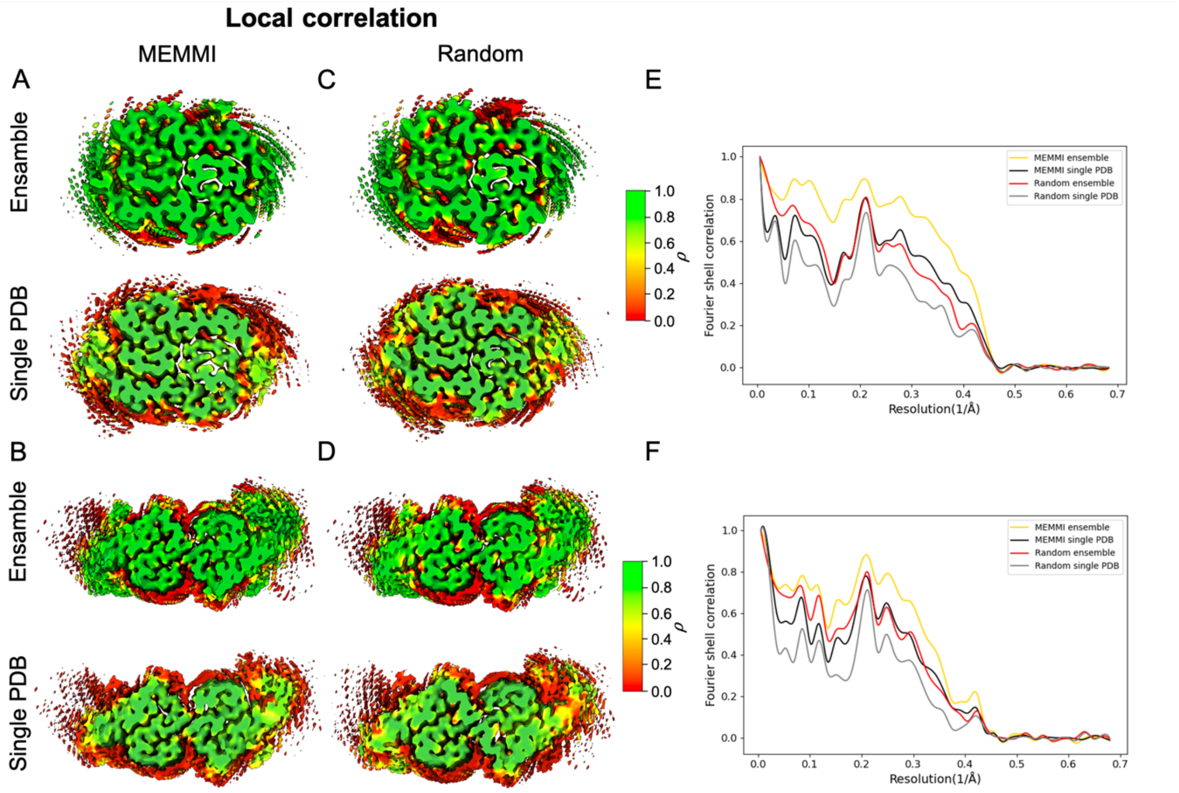

Figure S7. Comparison of local correlations and Fourier Shell Correlation (FSC) curves generated by single conformations versus ensembles. (A-D) Local correlation analysis between the experimental cryo-EM maps EMD-13800 **(A, C)** and EMD-13809 **(B, D)** with the cryo-EM maps back-calculated from the respective MEMMI ensemble (top) and a single MEMMI conformation (bottom) (A,B), as well as from the random ensemble (top) and a single random conformation (bottom) (C, D). **(E-F)** FSC curves for type I (E) and type II (F) filaments comparing density maps back-calculated from the MEMMI ensemble (yellow), a single MEMMI conformation (black), the random ensemble (red), and a single conformation from the random ensemble (gray).

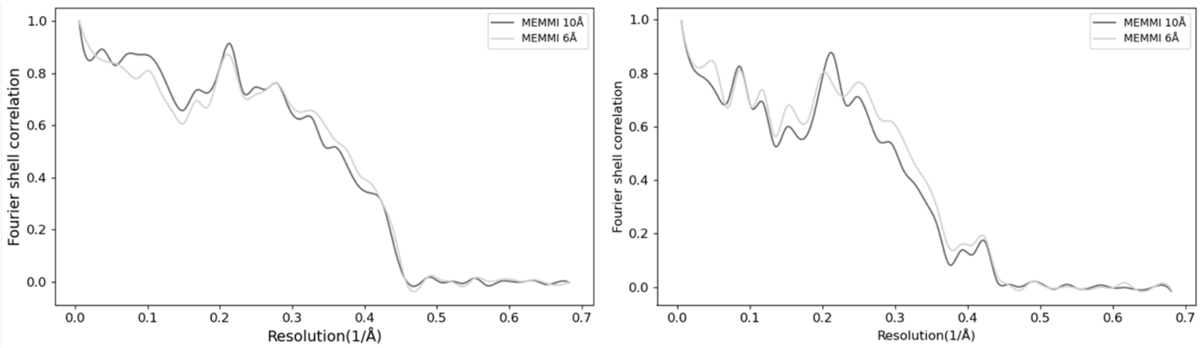

Figure S8. Comparison of FSC curves MEMMI density maps obtained from simulations with different segmentation cutoffs. The FSC curve of the back-calculated density map from the ensemble generated using the experimental cryo-EM map, trimmed at 6Å around the atomic model, is shown in light gray, while the curve for a 10Å cutoff is displayed in dark gray. Type I filament (left panel) and type II filament (right panel).

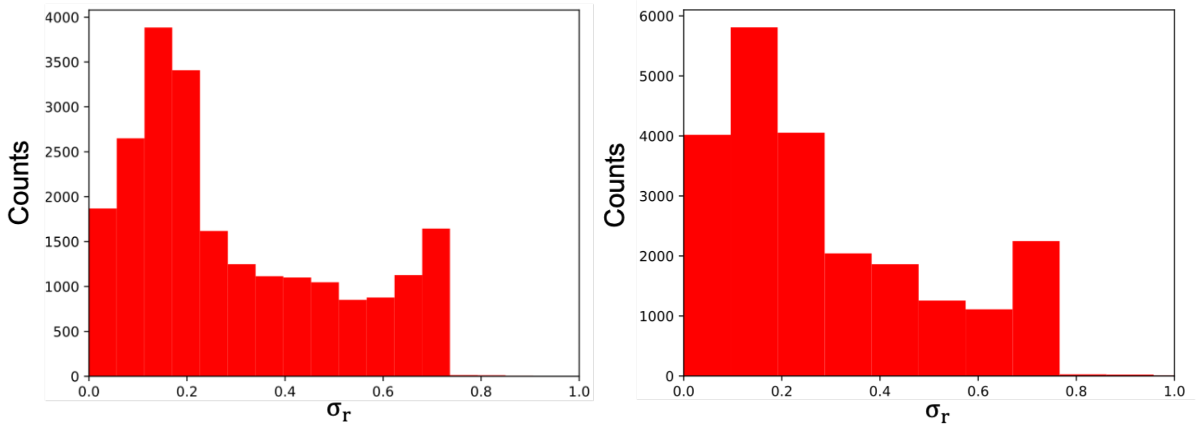

Figure S9. Relative errors in the data from the MEMMI simulations. Histogram of the error in the GMM data for the type I filament (left panel) and type II filament (right panel) as obtained by the respective structural ensemble.

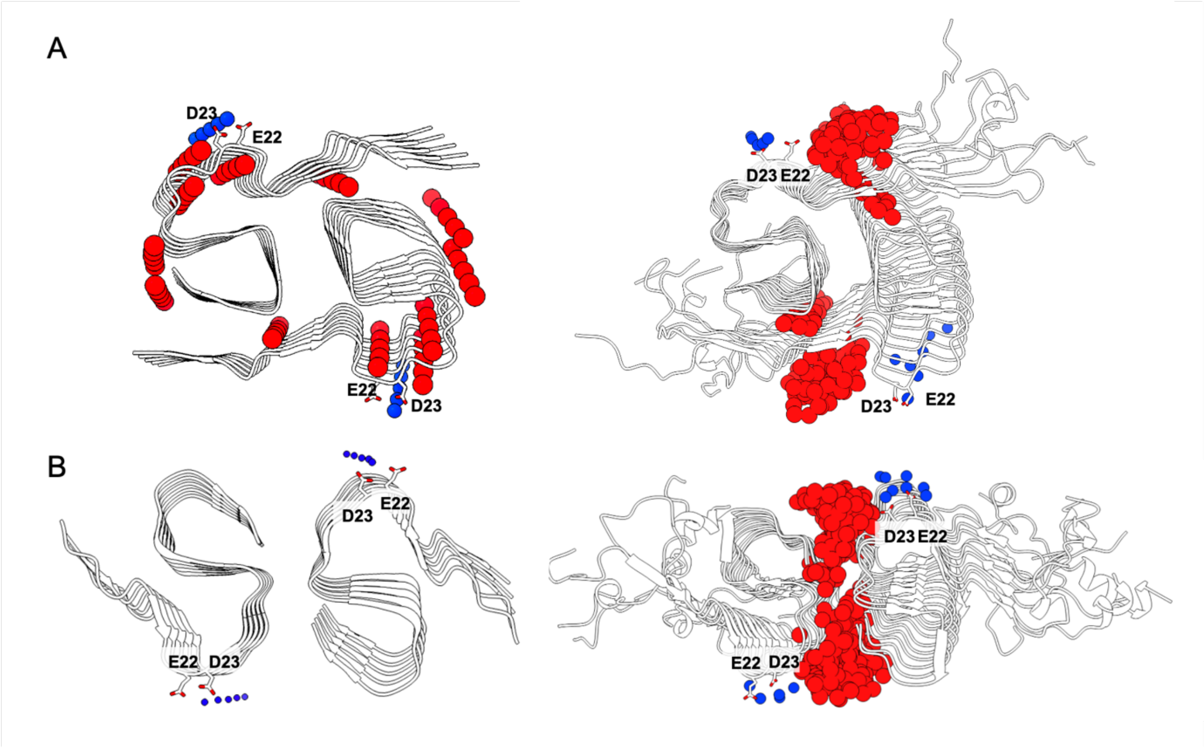

Figure S10. Comparison of ordered molecules from deposited PDB structures (7Q4B and 7Q4M) with those identified in our solvent diffusion analysis. (A-B) Schematic representation of the type I filament (A) and type II filament (B). On the left, the deposited PDB structures are shown, and on the right, the MEMMI conformations are presented, both including their respective ordered solvent molecules. The protein is shown in gray, ordered water molecules are in red, unknown non-protein atoms in the PDB structures are shown in blue, and sodium ions in the MEMMI representation are also shown in blue.

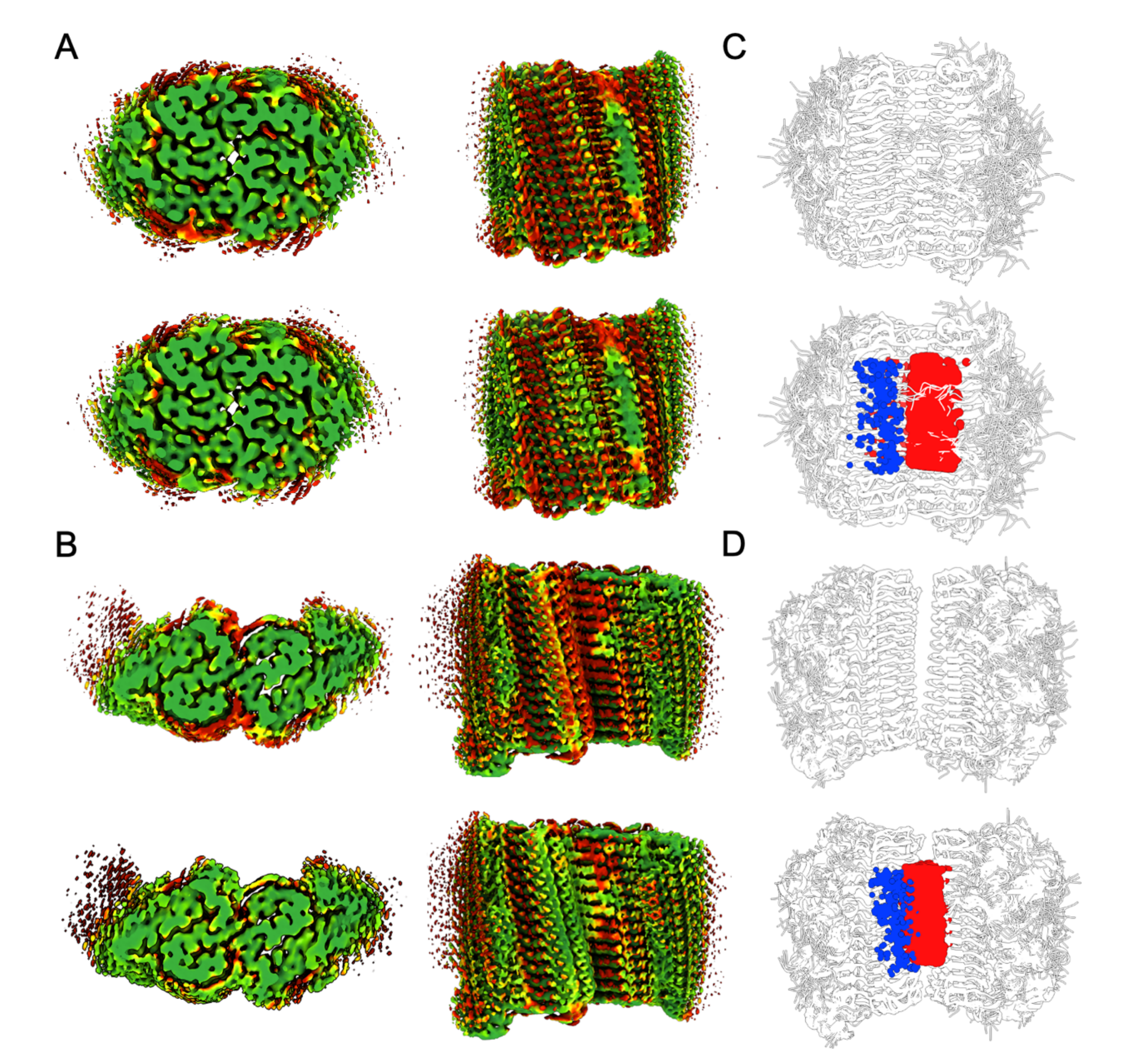

Figure S11. Impact of ordered molecules on local correlation analysis. (A-B) Local correlation analysis between the experimental cryo-EM maps EMD-13800 (A) and EMD-13809 (B) and the cryo-EM maps back-calculated from the respective MEMMI ensemble (top) and from the MEMMI ensemble including ordered water and ion molecules identified in the solvent diffusion analysis (bottom). **(C-D)** Schematic representation of the ensemble used for the back-calculation of the type I filament (C) and type II filament (D). In the upper panels, the protein ensemble is shown in gray, while the lower panels also display the ordered water molecules in red and the sodium ions in blue.
